# World Health Organization (WHO) Grade 1 Astrocytoma in a Female From Rural India: A Case Report

**DOI:** 10.7759/cureus.50554

**Published:** 2023-12-15

**Authors:** Mohammed Ibtesam A Siddiqui, Pratap Parihar, Gaurav V Mishra, Anshul Sood, Keyur Saboo

**Affiliations:** 1 Department of Medicine, Datta Meghe Institute of Higher Education and Research, Wardha, IND; 2 Department of Radiodiagnosis, Datta Meghe Institute of Higher Education and Research, Wardha, IND

**Keywords:** who grade 1, mri, astrocytoma, temporo-parietal, case report

## Abstract

Astrocytomas are rare in adults and less common in the parietal and temporal regions of the brain parenchyma. The current case is of a 26-year-old female patient who presented with a four-month history of headaches and a two-month history of vomiting. The patient's MRI brain showed an ill-defined, thick-walled lesion in the right parietal and temporal region with mass effect, which on histopathology confirmed to be a case of WHO Grade 1 astrocytoma. This manuscript describes the imaging and histopathological appearance of WHO Grade 1 astrocytoma in an adult female.

## Introduction

Astrocytomas are tumors that form in the central nervous system (CNS) from astrocyte cells, which have the shape of stars. There are four grades of astrocytoma; World Health Organization (WHO) Grade 1 (pilocytic astrocytoma), Grade 2 (diffuse astrocytoma), Grade 3 (anaplastic astrocytoma), and Grade 4 (glioblastoma) [1].

Children and teens are most frequently affected by WHO grade 1 astrocytomas and are more common in males, who account for 63% of cases worldwide [1].

The common locations for Grade 1 astrocytoma include the cerebellum, spinal cord, and optic pathway [2]. The present case is of an adult female patient who presented to us with complaints of headache and several episodes of vomiting and was found to have an ill-defined, thick-walled lesion in the right parietal and temporal region with a mass effect on MRI which was histo-pathologically proven to be a WHO Grade 1 astrocytoma.

## Case presentation

The current case is of a 26-year-old female who came to the emergency department with complaints of headaches for four months and vomiting for two months. The headache was diffuse, predominantly on the right temporal region, throbbing, and severely intense. The patient had to leave all her work when the pain started. There were no aggravating factors, and she had to take painkillers to relieve the headache. Initially, the headache was not frequent and would occur once a week, but it has increased to almost once in two days. The vomiting was projectile in nature and was always associated with headache.

The patient was referred to the neurology outpatient department for further management. She had no history of loss of consciousness or visual disturbance, ear, nose, and throat bleeding, or head trauma. She had normal power in both the upper and lower extremities on examination. She was advised to take blood tests and an MRI of the brain with contrast to rule out any organic cause for the above-mentioned complaints.

There was an increase in the white blood cell (WBC) count and urea; the rest of the blood tests were unremarkable (Table 1).

**Table 1:** Lab parameters of the patient. MCHC: mean corpuscular hemoglobin concentration; MCV: mean corpuscular volume; MCH: mean corpuscular hemoglobin; RBC: red blood cell; WBC: white blood cell; HCT: hematocrit; RDW: red cell distribution width; APTT: activated partial thromboplastin time; INR: international normalized ratio; HPF: high power field.

Investigation	Patient	Reference values
Hemoglobin	12.3	12.1-15.1g/dl
MCHC	34.1	32-36g/dl
MCV	86.2	80-100fl
MCH	29.4	27-33pg
Total RBC count	3.2	3.8 -5.2×10*12L
Total WBC count	24100	4000-9000g/dl
Total platelet count	190000	150000-400000 g/dl
HCT	27.6	36-44%
Granulocytes	85	1.5-8.5 ×10^9/L
RDW	13.6	12.2-16.1%
APTT patient	29.9	21-35 seconds
Prothrombin time patient	12.7	10-13 seconds
INR	1.07	<=1.1
Epithelial cells	1-2 CELLS/HPF	15-20 CELLS/HPF
Pus cells	1-2 CELLS/HPF	5-7 CELLS/HPF
Urea	47	5-20 mg/dl
Creatinine	0.9	0.6-1.1 mg/dl

**Figure 1:**
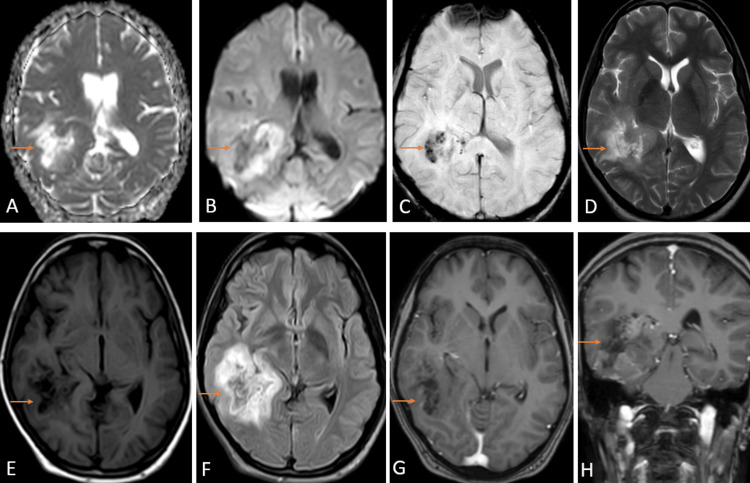
MRI brain with contrast axial section of ADC (A), DWI (B), SWI (C), T2WI (D), T1WI (E), FLAIR sequence (F), post contrast T1WI (G), and post contrast T1WI coronal section (H) showing an ill-defined, thick-walled, intraaxial, minimally enhancing altered signal intensity lesion with perilesional edema in the subcortical and deep white matter with overlying thickened cortex in the right parietal and temporal region. The lesion shows peripheral restriction on DWI with corresponding low signal intensity on ADC, appearing heterogeneously hyperintense on T2WI and showing T2IW/FLAIR mismatch, hypointense with small cystic areas on T1WI with few areas of blooming on SWI. DWI: diffusion-weighted imaging; ADC: apparent diffusion coefficient; T2WI: T2 weighted imaging; T1WI: T1 weighted imaging; FLAIR: fluid attenuation inversion recovery; SWI: susceptibility-weighted imaging.

MRI Brain with contrast revealed an ill-defined, thick-walled, intra-axial, minimally enhancing altered signal intensity lesion with perilesional edema in the subcortical and deep white matter. The lesion showed peripheral restriction on diffusion-weighted imaging (DWI) with corresponding low signal intensity on apparent diffusion coefficient (ADC), appearing heterogeneously hyperintense on T2 weighted imaging (T2WI) and showing T2WI/fluid attenuation inverse recovery (FLAIR) mismatch, hypointense with small cystic areas on T1 weighted imaging (T1WI) with few areas of blooming on susceptibility-weighted imaging (SWI) (Figure 1).

**Figure 2:**
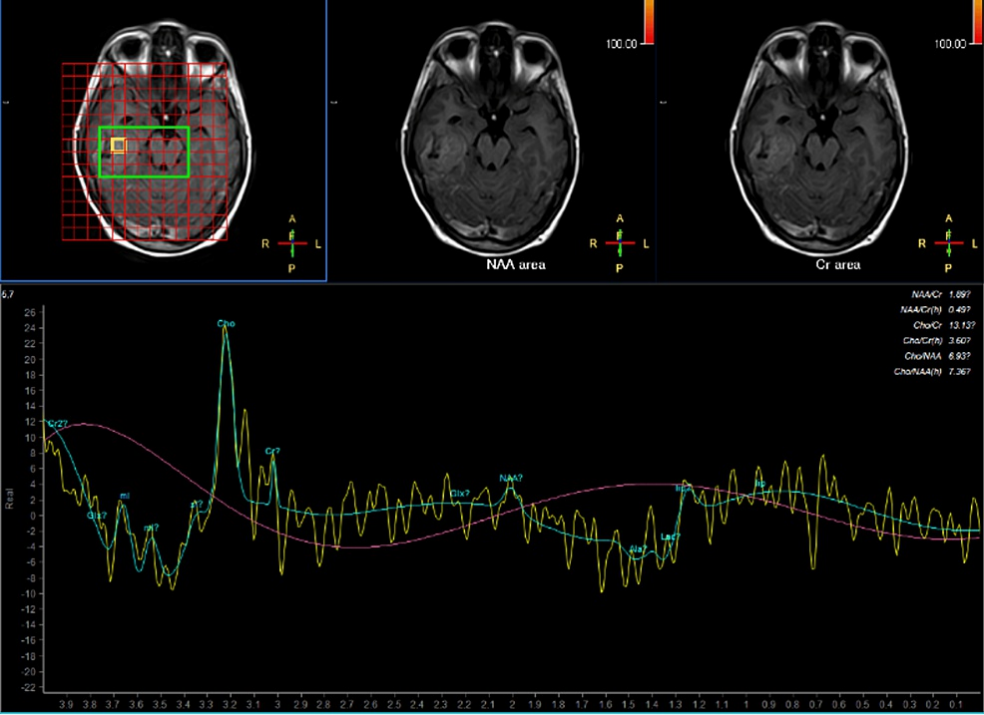
MR spectroscopy showing an increased choline peak, decreased NAA peak, decreased choline : NAA, and lack of lactate peak at 1.3 ppm. MR: magnetic resonance; NAA: N-acetyl aspartate.

On magnetic resonance (MR) spectroscopy, there was an increased choline peak, decreased N-acetyl aspartate (NAA) peak, decreased choline : NAA, and lack of lactate peak at 1.3 ppm (Figure 2).

**Figure 3:**
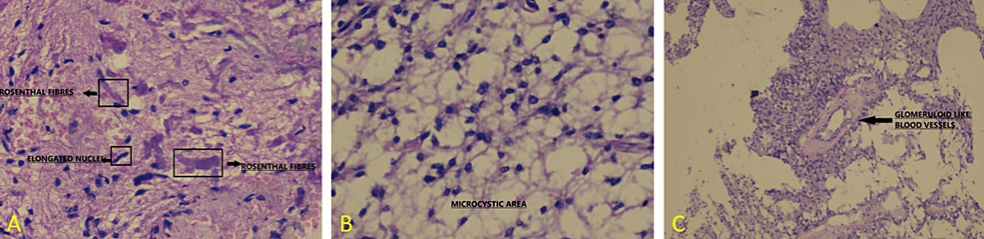
Histopathological images (A), (B), and (C) showing elongated nuclei, bipolar piloid processes, and pink Rosenthal-like structure with loose microcystic area having round to oval nuclei and eosinophilic granular bodies-like structure. Small glomeruloid-like blood vessels are also seen.

The patient underwent right parieto-temporal craniotomy and excision of the lesion. The histopathology report confirmed pilocytic astrocytoma (WHO Grade 1) (Figure 3).

Post-operative CT was done satisfactorily. The patient was managed conservatively. She was conscious, oriented, and vitally stable and was discharged three days after the operation with a follow-up plan after 10 days or in case of emergency.

## Discussion

Grade 1 astrocytomas are the primary brain tumors most frequently diagnosed in children and adolescents [1]. The risk factors directly leading to the development of pilocytic astrocytomas are still unclear and mostly idiopathic [3]. The patient may present with the symptoms of elevated intracranial pressure (headache, nausea, and vomiting) as was in our case. Ataxia and cranial nerve involvement are also frequently associated with the disease [3].

Other common locations for astrocytomas include the hypothalamus, optic pathways, or brainstem. When located in the hypothalamus, the tumors may cause endocrine disturbances and might lead to diabetes insipidus, precocious puberty, or electrolyte imbalance. When present in the optic pathways, the tumor may cause loss of visual acuity or field abnormalities [4]. Optic nerve tumors are reported to be linked to radiotherapy, while brainstem tumors are related to chemotherapy [5].

The classification of these malignancies has been furthered by new diagnostic criteria based on histology and molecular characterization with isocitrate dehydrogenase (IDH) mutation status and 1p/19q codeletion status, adding new insights into prognosis and therapeutic response [6]. However, in line with the WHO, astrocytomas, oligodendrogliomas, mixed oligoastrocytomas, and ependymal tumors are the four primary categories of gliomas. These are further broken down into WHO Grades I through IV based on histological distinctions and cellularity, mitotic activity, atypical nuclei, microvascular proliferation, and necrosis based on cytology and histology [7].

One should be aware of the association of astrocytomas with many syndromes like Cowden, Turcot, Lynch, Li-Fraumeni, and neurofibromatosis type I [3].

The treatment of choice is surgical excision, and currently, a cutting-edge microsurgical technology is being used concurrently [8]. With survival rates close to 7 to 8 years following surgery, it is proven to be advantageous for low-grade cancers [9].

## Conclusions

In summary, this case report presents a truly unusual occurrence, a Grade 1 astrocytoma in an adult female. Such cases are exceedingly rare, challenging our conventional understanding of the demographics of astrocytoma incidence. This unique presentation underlines the importance of maintaining a broad diagnostic perspective and adaptability in the face of atypical clinical profiles. The successful management of this Grade 1 astrocytoma in an adult female reinforces the idea that individualized treatment plans are essential. While considered routine for such tumors, surgical intervention highlights the potential for positive outcomes.

The exceptional nature of this case prompts the need for further investigation into the underlying factors contributing to these occurrences in this specific demographic. Ongoing research is vital in understanding the epidemiology and behavior of these tumors. In conclusion, this case report serves as a testament to astrocytomas' remarkable and uncommon aspects. It reminds us that even well-established medical knowledge can be challenged and that ongoing research is crucial to adapt to the unexpected variations in neuro-oncology.
